# Blood-Brain Barrier Dysfunction and the Pathogenesis of Alzheimer’s Disease

**DOI:** 10.3390/ijms18091965

**Published:** 2017-09-13

**Authors:** Yu Yamazaki, Takahisa Kanekiyo

**Affiliations:** Department of Neuroscience, Mayo Clinic, 4500 San Pablo Road, Jacksonville, FL 32224, USA; yamazaki.yu@mayo.edu

**Keywords:** amyloid-β, astrocytes, basement membrane, cerebral amyloid angiopathy, endothelial cells, neurovascular unit, pericytes, tight junctions

## Abstract

Brain capillary endothelial cells form the blood-brain barrier (BBB), which is covered with basement membranes and is also surrounded by pericytes and astrocyte end-feet in the neurovascular unit. The BBB tightly regulates the molecular exchange between the blood flow and brain parenchyma, thereby regulating the homeostasis of the central nervous system (CNS). Thus, dysfunction of the BBB is likely involved in the pathogenesis of several neurological diseases, including Alzheimer’s disease (AD). While amyloid-β (Aβ) deposition and neurofibrillary tangle formation in the brain are central pathological hallmarks in AD, cerebrovascular lesions and BBB alteration have also been shown to frequently coexist. Although further clinical studies should clarify whether BBB disruption is a specific feature of AD pathogenesis, increasing evidence indicates that each component of the neurovascular unit is significantly affected in the presence of AD-related pathologies in animal models and human patients. Conversely, since some portions of Aβ are eliminated along the neurovascular unit and across the BBB, disturbing the pathways may result in exacerbated Aβ accumulation in the brain. Thus, current evidence suggests that BBB dysfunction may causatively and consequently contribute to AD pathogenesis, forming a vicious cycle between brain Aβ accumulation and neurovascular unit impairments during disease progression.

## 1. Introduction

Blood vessels are the essential components of the circulatory system that transport blood throughout the body, proper functioning of which is critical to maintaining the homeostasis of organs and tissues. They deliver oxygen and nutrients, remove metabolic waste, and mediate signaling of the endocrine glands as well as provide a way for tissue to interact with the peripheral immune system [1,2,3,4]. Vasculatures are composed of different segments, including arteries, arterioles, capillary beds, venules, and veins, all of which differ from each other structurally and functionally. Furthermore, these vascular segments—particularly microvessels—have unique properties, depending on their corresponding organs or tissues and how they respond to specific requirements [5]. In the central nervous system (CNS), capillary endothelial cells form the blood-brain barrier (BBB), which precisely controls the entry of blood components, including plasma proteins, ions, red blood cells, and leukocytes, into the CNS, as well as the elimination of toxic molecules to the blood [5,6,7]. Because the BBB plays a critical role in maintaining CNS homeostasis, the disturbance of proper BBB functioning is increasingly recognized as a potential contributor in a number of neurological disease pathogeneses, including late-onset Alzheimer’s disease (AD) [8,9,10].

AD is the most common cause of dementia in the elderly, and is estimated to affect approximately 14 million people in the United States by 2050 [11,12]. Pathologically, AD is characterized by extracellular amyloid-β (Aβ) deposition in brain parenchyma as senile plaques and in vessels as cerebral amyloid angiopathy (CAA) [13,14]. AD is also characterized by a neuronal accumulation of phosphorylated tau–forming neurofibrillary tangles, which are typically accompanied by neuronal loss and glial activation [15,16,17]. While these are the central pathological hallmarks in AD brains, the majority of AD cases have been shown to exhibit a complex combination of multiple pathologies [18]. In particular, some extents of vascular pathology are frequently detected in AD brains. In one study, more than 77% (316/410 cases) of postmortem AD brains had circle of Willis atherosclerosis, whereas the prevalence was significantly higher than that of control individuals (47%, 28/59 cases) [19]. Furthermore, other types of vascular pathologies such as infarcts, microbleeds, and white matter changes also often exist in AD patients [18]. In view of the increasing interest in cerebrovascular contributions to AD pathogenesis and BBB function in maintaining CNS homeostasis, our review summarizes current evidence for BBB alteration during AD progression, and discusses how BBB dysregulation is associated with disease pathogenesis.

## 2. Blood-Brain Barrier (BBB) in the Neurovascular Unit

**Figure 1:**
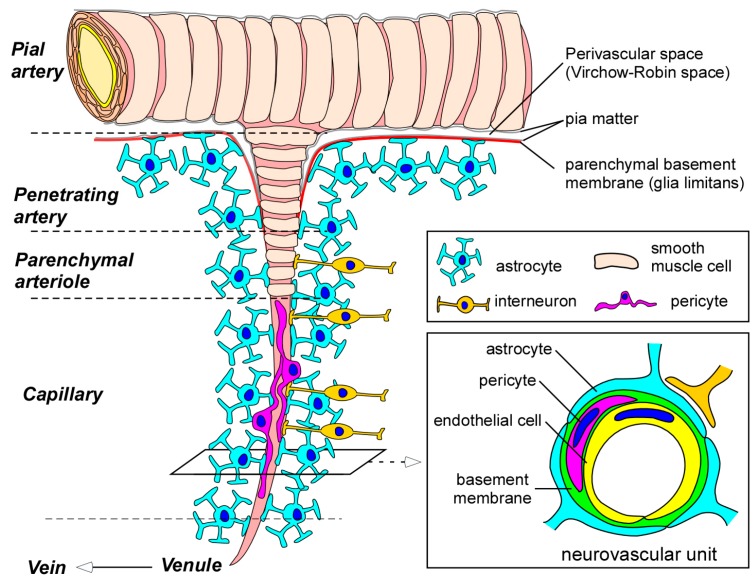
Blood-brain barrier (BBB) and the neurovascular unit. Pial arteries branch out into smaller arteries called penetrating arteries. The penetrating arteries go further down into the brain parenchyma, giving rise to parenchymal arterioles, which eventually branch off into capillaries. Whereas pial and penetrating arteries are covered by vascular smooth muscle cells and are separated from brain tissues by the parenchymal basement membrane (glia limitans), parenchymal arterioles and capillaries become associated with neurons and astrocytes. Parenchymal arterioles are covered by one layer of smooth muscle cells. In capillaries, endothelial cells form the BBB. BBB properties in endothelial cells are further maintained and regulated through communications with basement membranes and other neighboring cells in neurovascular unit such as pericytes, astrocytes, and interneurons. BBB indicates blood-brain barrier.

In brain capillaries, endothelial cells form the tube structure with barrier integrity, in which the abluminal surface is covered by basement membranes composed of extracellular matrix. The endothelial tubes are surrounded by pericytes, astrocyte end-feet, and neurons, comprising the neurovascular unit (Figure 1). While physical barrier structures in endothelial cells predominantly control BBB integrity, molecular barrier systems through endothelial transporters can mediate the influx and efflux of specific molecules at the BBB. Furthermore, other cell types and basement membranes in the neurovascular unit are also critical for the induction and maintenance of the proper functioning of the BBB [5,7,8,9,10].

### 2.1. Endothelial Cells

**Figure 2:**
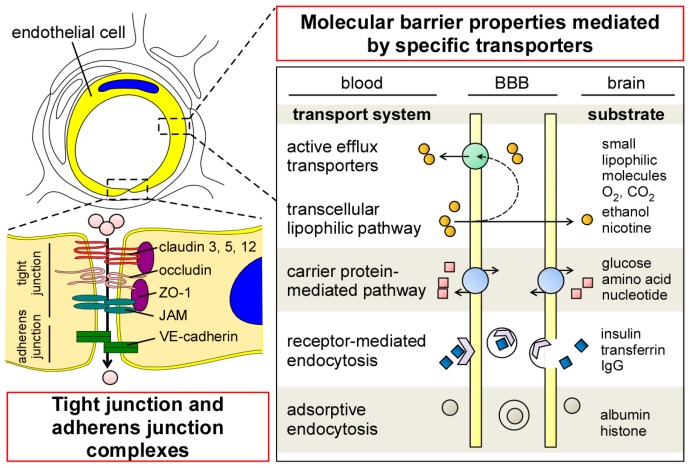
Physical and molecular properties of endothelial cells contributing to BBB integrity and function. Tight junction and adherens junction complexes between endothelial cells restrict paracellular flux across the BBB. In addition, some nutrients and essential molecules are selectively transported from luminal to abluminal membranes by specific influx transport systems. Most of the small lipophilic molecules passively diffused across the lipid bilayer are returned to the blood by ATP-dependent efflux transporters. ZO-1 (zonula occludens-1); JAM (junctional adhesion molecule); VE-cadherin (vascular endothelial-cadherin); lgG (immunoglobulin G).

To ensure the precise regulation of transport across the BBB, endothelial cells in the CNS have several unique properties compared to those in the periphery. Although endothelial cells in the CNS have no fenestrations, they form tight junctions (TJs) that limit paracellular permeability between the luminal and abluminal compartments [5,20] A series of transmembrane proteins (e.g., claudin, occludin, and junctional adhesion molecule (JAM)) are involved in constructing TJs at the BBB (Figure 2). Claudin is the major structural component of the TJs, and is a tetraspan transmembrane protein composed of 207–305 amino acids in humans [21]. Indeed, overexpression of claudins sufficiently induces TJ strands in fibroblasts [22], whereas their disruption compromises the paracellular barrier integrities in kidney cells [23,24,25,26]. While different isoforms of claudin are expressed in endothelial/epithelial barriers both in the CNS and periphery, the distributions of claudin-1, -3, -5, and -12 have been identified in brain endothelial cells [27,28,29,30]. In particular, claudin-5 is highly expressed in brain endothelial cells [28], where its deficiency results in the loosening of the BBB in a mouse model [31]. Occludin is also a tetraspan transmembrane TJ protein which possesses 522 amino acids [32]. While the disruption of occludin decreases barrier integrities in vitro [33,34], BBB alteration has not been detected in occludin-deficient mice [35]. Those transmembrane TJ proteins are connected to the actin cytoskeleton through Zonula occludens-1 (ZO-1), a member of membrane-associated guanylate kinase–like (MAGUK) protein [36,37] The deletion of ZO-1 leads to TJ disruption and the redistribution of active myosin II in vitro [38]. Together, these restrictive TJ structures in brain capillary endothelial cells reduce paracellular diffusion and limit transcellular activity, thereby strictly regulating the nonspecific influx/efflux of biological molecules across the BBB. In addition to TJ, the dynamic opening and closure of the cell-to-cell adherens junction also regulates BBB permeability [39]. Vascular endothelial (VE)-cadherin is an endothelial-specific molecule located at the adherens junction, and plays an important role for the control of endothelial permeability and leukocyte extravasation at the BBB [40].

In cerebrovascular endothelial cells, transcytosis activity is known to be extremely low compared to that in peripheral endothelial cells [41,42]. The low rate of transcytosis likely restricts the transcellular movement of macromolecules by vesicles. Nonetheless, several specific molecules can be transported across the endothelial barrier through the transcellular lipophilic pathway, carrier protein–mediated pathway, receptor-mediated endocytosis, and adsorptive endocytosis (Figure 2) [20,43]. Transendothelial passive diffusion allows the influx of small, nonpolar, and lipophilic molecules into brains across the lipid bilayer of endothelial cells [20], while most of them are likely eliminated to the blood through ATP-dependent efflux transporters [43,44]. Glucose, hormones, amino acids, and nucleotides can pass through the BBB by the carrier-mediated transport [43]. Since the gradient in concentration across the BBB is the major driving factor for carrier-mediated transport, the pathway is likely affected by the size, affinity, and physiochemical properties of each specific molecule [45]. In addition, the receptor-mediated or adsorption-mediated endocytic transport system enables several large molecules such as proteins and peptides to be delivered into the brain across the BBB. The unique cellular phenotypes in cerebrovascular endothelial cells have been represented by the enrichment of genes coding transporters in transcriptome, which accounts for more than 10% of gene expressions in the cell type [46].

While the entry of neutrophils and lymphocytes from blood into tissues are limited under homeostatic conditions, activated endothelial cells increase the expression of leukocyte adhesion molecules (LAMs), which triggers invasion of those cells [5]. Given that endothelial cells in the CNS express extremely low levels of LAMs compared with those in peripheral tissues [47,48,49], the property may prevent the excess entry of immune cells from blood to brain parenchyma under homeostatic conditions, contributing to the immunologic privilege in the CNS [50].

### 2.2. Pericytes

Pericytes are mural cells covering the abluminal surface of microvessels. In the neurovascular unit, pericytes are embedded in a thin layer of basement membrane which separates pericytes from endothelial cells and end-feet of astrocytes (Figure 1). While most of the pericyte bodies and processes do not attach with endothelial cells because of the basement membrane, interdigitations of pericyte and endothelial cell membranes can directly connect in the area lacking basement membrane, forming the peg-and-socket connections. In addition, adherens junctions and gap junctions, which are regulated by *N*-cadherin and connexin 43, respectively, allow pericytes to communicate with endothelial cells [51,52]. Pericytes have been shown to regulate angiogenesis, extracellular matrix formation, and BBB functioning in developing brains as well as adult brains [52,53,54,55,56,57]. In addition, the contractile property of pericytes contributes to the regulation of blood flow by controlling capillary diameter [52,58,59]. Highlighting the unique properties of brain pericytes, pericytes are much more abundant in the CNS than in peripheral tissues; the number of pericytes is equal to that of endothelial cells in the brain, whereas it is estimated to be only around 1% and 10% of the number of endothelial cells in peripheral striated muscles and lung, respectively [60].

### 2.3. Astrocytes

Astrocytes, the main class of glial cells, are star-shaped cells with many processes emanating from the cell body [61]. Astrocytes surround most portions of the microvessels and capillaries and interact with endothelial cells through the end-feet of their processes in the neurovascular unit (Figure 1) [61,62]. Furthermore, a single astrocyte can contact thousands of synapses, as well as capillaries, through their processes [63]. As such, astrocytes provide a cellular link between neuronal activity and blood vessels, termed neurovascular coupling. In addition to their roles providing structural, metabolic, and trophic support for neurons [64,65], astrocytes play critical roles in regulating cerebral blood flow in response to neuronal activity by relaying signals [5,66,67]. Astrocytes also participate in maintaining BBB function by inducing barrier properties and the polarization of transporters [62,68,69,70], while an in vivo study suggested that a functional BBB is already present during embryogenesis, even before astrocyte generation [54].

### 2.4. Basement Membranes

Basement membranes in the neurovascular unit also significantly contribute to BBB integrity through several mechanisms. The predominant constituents of the cerebrovascular basement membranes include collagen IV, laminin, perlecan, nidogen, and fibronectin, which are extracellular matrix proteins produced by each cell type in the neurovascular unit [71,72]. There are two types of basement membranes in the unit: (1) an endothelial basement membrane composed of extracellular matrix produced by endothelial cells and pericytes; and (2) a parenchymal basement membrane formed by those from astrocytes [73,74,75]. While the endothelial basement membrane is enriched in laminin α4 and α5 [76], laminin α1 and α2 isoforms are more abundant in the parenchymal basement membrane [73,74,77]. Basement membranes function as a physical barrier surrounding the abluminal surface of endothelial cells and anchor the cells in place at the BBB (Figure 1). In addition, they also contribute to BBB regulation, where the extracellular matrix mediates diverse signaling in endothelial cells and pericytes [75]. Indeed, basement membrane components have been shown to regulate the cellular localization of occludin in endothelial cells, thereby influencing barrier stability [78,79].

## 3. BBB Alteration in Alzheimer’s Disease (AD)

As described above, BBB integrity is strictly controlled by cells and basement membranes in the neurovascular unit in physiological conditions. However, the barrier function is likely compromised during aging and AD. In this section, we summarize and discuss the current evidence from clinical studies investigating BBB integrity in AD patients using biochemical and histological approaches in postmortem brains, cerebrospinal fluid (CSF) biomarkers, and brain imaging techniques.

### 3.1. Leakages of Blood-Derived Molecules in Postmortem AD Brains

The measurement of plasma- or serum-derived molecules in the brain parenchyma has been widely used as a method to detect BBB disruption. Perivascular immunoreactivities of plasma proteins, albumin, and IgG, have been detected in microvascular segments associated with senile plaques and CAA in AD brains [80,81]. In addition, increased levels of hemoglobin-derived peptides were identified by reverse phase HPLC (high performance liquid chromatography) in the cerebellum of patients with AD, compared to control cases with no significant neuropathology [82]. Elevated prothrombin amounts in AD postmortem tissues have also been shown by immunohistochemical analysis and ELISA (enzyme-linked immunosorbent assay), the degree of which was positively correlated with the Braak stage [83]. Together, these observations suggest the existence of BBB disruptions in AD brains. However, there are several studies showing conflicting results. Immunohistochemical staining for albumin, prealbumin, immunoglobulin, C1q, C3c, or fibrinogen failed to detect higher degrees of serum protein extravasation in AD brains than control individuals [84,85]. Differences in the procedures for sample preparation [86] and immunohistochemical staining may be potential factors leading to these inconsistent results. Moreover, the heterogeneity of concomitant vascular pathology in AD brains might also contribute to the discrepancy in these findings. Further studies in larger cohorts and optimizations for methodology are needed to determine whether parenchymal accumulation of peripheral blood-derived molecules is specifically exacerbated in AD brains, representing BBB disruption.

### 3.2. Cerebrospinal Fluid (CSF)/Blood Albumin Ratio in AD Patients

AD patients have been shown to possess an increased CSF/serum or CSF/plasma ratio of albumin, which is often used as a proxy for BBB disruption [87,88,89,90]. However, some studies reported that the change of CSF markers was evident only in AD patients with vascular risk factors or vascular lesions, but not in AD cases without them [91,92,93], even though the majority of AD cases may have some extent of vascular pathology. A meta-analysis of 31 clinical studies (1953 individuals) measuring the CSF/serum albumin ratio showed that the BBB permeability parameters are increased in association with aging and vascular dementia, but to a lesser degree with AD and white matter lesions [94]. Thus, vascular pathology, rather than senile plaque deposition or tauopathy, may impact the CSF/serum albumin ratio in AD. Thus, BBB disruption in AD patients should be interpreted by taking the degree of concomitant vascular factors into consideration. In addition, because AD patients have demonstrated disturbed turnover of CSF proteins [95,96], the albumin ratio of CSF/plasma may not precisely represent BBB permeability in AD.

### 3.3. Evaluation of BBB Function through Brain Imaging in AD Patients

Earlier brain imaging studies using computed tomography (CT) [97,98] and [^68^Ga]-EDTA (ethylene diamine tetra acetic acid) positron emission tomography (PET) [99] failed to show an increase in permeability in AD patients, although the number of cases analyzed was small. A study using dynamic contrast-enhanced magnetic resonance imaging (MRI) through gadolinium-diethylenetriamine pentaacetic acid (Gd-DTPA) injection suggested the enhanced BBB permeability in AD patients, compared to healthy control individuals, as higher levels of Gd-DTPA drainage into the CSF was detected in AD cases, whereas there was no overall difference in the extent of leakage into brain parenchyma [100]. A recent study using an advanced dynamic contrast-enhanced MRI protocol showed an age-dependent increase in BBB permeability in the entire hippocampus, CA1 region, and dentate gyrus [101]. Furthermore, the increase in BBB permeability in these regions was evident in patients with mild cognitive impairment (MCI) compared to aged cognitive healthy patients [101]. As such, recent advances in brain imaging technology might allow us to further investigate BBB integrity in AD patients.

## 4. Neurovascular Unit Dysregulation and AD

While further clinical studies are needed to assess BBB function in AD patients, increasing evidence from in vitro and in vivo studies suggests the disturbance of the neurovascular unit in AD. In this section, we summarize how each of the components in the neurovascular unit is affected by the presence of AD-related pathology. We also discuss how these changes in cellular properties could contribute to AD pathogenesis.

### 4.1. Endothelial Cell Alternation in AD

In postmortem human brains, TJ proteins, occludin, claudin-5, and ZO-1 were substantially reduced in capillaries with CAA, which was accompanied by increased fibrinogen leakages in the brain parenchyma [102,103]. In addition, alterations in cerebral TJs were also observed in amyloid model 5XFAD mice. Electron microscopy demonstrated that lengths of TJs in 5XFAD mice were significantly shorter than those in littermate control mice [104]. Consistent with those findings, the exposures of Aβ42, in particular that with the oligomeric form, significantly decreased levels of occludin, claudin-5, and ZO-1 [104,105] and compromised the barrier integrity [105] in a murine brain bEnd.3 endothelial cell line. Other reports also showed the reduction of occludin by administrations with Aβ40 and Aβ42 in human brain endothelial hCMEC/D3 cells [106] and primary rat brain endothelial cells [107], respectively. Furthermore, exogenous application of Aβ42 likely downregulates the JAM (an integral membrane protein at the TJ) in human umbilical vein endothelial cells (HUVECs) [108], although the lack of specific barrier properties in HUVECs suggests that these cells are a less suitable model for extrapolating the findings to brain microvessels [109]. In addition, hyperhomocysteinemia has been shown to induce the significant decrease of VE-cadherin in cerebrovasculature and exacerbated BBB permeability, as well as increased cerebrovascular deposition of Aβ and fibrinogen in a mouse model [110]. Thus, Aβ is likely to disrupt the organization of TJs and adherens junction in endothelial cells, thereby disturbing their barrier function.

While glucose transporter 1 (GLUT1) is a type 3 integral transmembrane protein specifically expressed in endothelial cells in the brain, GLUT1 is significantly reduced in the brain microvessels of AD patients and amyloid mouse models [111,112,113,114,115]. Of note, endothelial GLUT1 deficiency initiates early BBB disruption as represented by the reduction in TJ proteins and extravascular accumulation of fibrinogen and IgG in mice [116]. Furthermore, GLUT1 deficiency also leads to cerebral microvascular degeneration followed by the accelerated Aβ pathology in an amyloid mouse model [116]. Thus, the reduction in GLUT1 in microvessels during AD could contribute to disease pathogenesis. However, since defective GLUT1 causes hypoglycorrhachia, seizures, and developmental delay [117,118,119], it remains unclear whether BBB dysregulation is a central mechanism initiating disease progression in GLUT1-related AD pathogenesis.

### 4.2. Cerebrovascular Pericyte Degeneration in AD

Through a communication with neighboring endothelial cells and astrocytes in the neurovascular unit, pericytes play multiple roles in the CNS, including a regulation of BBB integrity and clearance of metabolites [120]. In AD brains, coverages of microvessels by pericytes were significantly reduced, correlating with BBB disruption [121]. While age-dependent BBB breakdown in the hippocampus was reported in an antemortem study, patients with MCI showed a higher degree of BBB permeability, which is associated with an increased soluble platelet-derived growth factor receptor β (PDGFRβ) in the CSF, representing pericyte damages [101]. Indeed, a mouse model with pericyte deficit has been shown to lead to age-dependent BBB disruption, leading to secondary neurodegeneration [57]. Moreover, pericyte deficiency causes accelerated brain Aβ deposition and CAA formation with impaired clearance of soluble Aβ40 and Aβ42 from brains in an amyloid mouse model [122]. Thus, reduced coverage in pericytes in AD may further exacerbate parenchymal and vascular Aβ accumulation.

### 4.3. Altered Perivascular Astrocytic End-Feet in AD

During the progression of AD and CAA, astrocyte characteristics are distinctly changed in postmortem human brains [121,123,124,125,126] and amyloid mouse models [114,126,127]. In the temporal cortex from AD patients, the reduced mRNA expressions of astrocytic end-feet water channel aquaporin 4 (AQP4) and activated astrocyte marker glial fibrillary acidic protein (GFAP) were observed in association with the severity of CAA pathology [126]. While global immunoreactivity of AQP4 was increased during aging and AD in the frontal cortex, perivascular AQP4 localization was significantly reduced in AD cases independent of age, compared to cognitively healthy individuals [125]. Loss of perivascular AQP4 localization was associated with a high degree of AD-related pathology, including Aβ burden and Braak stage [125]. In an amyloid mouse model, retraction and swelling in astrocytic end-feet was also observed in those surrounding parenchymal Aβ deposits and CAA in both early- and late-stage animals [114]. In addition, the redistribution of AQP4 from astrocytic end-foot membranes to non–end-foot membrane domains was detected in amyloid model mice, which is likely due to astrocyte depolarization induced by brain Aβ deposition [127]. Of note, paravascular astroglial water transport mediated by AQP4 not only supports CSF flux into the parenchyma but also facilitates the solute clearance through bulk interstitial fluid (ISF) drainage [128]. AQP4 deficiency has been shown to impair the clearance of [^125^I]-Aβ40 as well as [^3^H]-mannitol or [^3^H]-dextran-10 from the brain when injected into mouse brain parenchyma [128]. Thus, the dysfunction of astrocytic end-feet during AD progression may exacerbate Aβ accumulation by disturbing cerebrovascular Aβ clearance along the ISF drainage pathway.

### 4.4. Cerebrovascular Basement Membrane Pathology in AD

The thickening of basement membranes is likely one of the common pathologies detected in the brain capillaries of AD patients [129]. Immunohistochemical analyses have revealed that basement membrane components, including collagen IV, perlecan, and fibronectin, were increased in the frontal and temporal cortex from subclinical AD (Braak stage III–IV) and AD patients (Braak stage V–VI) compared to controls, whereas no significant difference was detected between subclinical AD and AD cases [130]. The extent of collagen IV staining was not associated with the severity of CAA in the frontal or occipital cortex from AD patients [131]. Western blotting showed increased total collagen and collagen IV levels in cerebral microvessels isolated from AD patients compared to those from controls [132], although there is a conflicting study reporting reduced collagen IV and elevated collagen I and III in AD vessels [133].

A mouse model demonstrated that vascular basement membranes play a critical role as pathways for the lymphatic drainage of ISF from the brain parenchyma to cervical lymph nodes, as well as the glymphatic drainage of CSF into ISF [134]. When gold nanoparticles or Aβ are injected into mouse brain parenchyma, they flow through the ISF drainage pathway along a basement membrane layer between endothelial cells and pericytes to the surrounding smooth muscle cells in the tunica media. On the other hand, the tracer injected into the CSF enters the brain through a lymphatic pathway along the basement membrane between the pia mater and glia limitans [134]. Importantly, the clearance of Aβ injected into the mouse hippocampus through the ISF drainage pathway has been shown to be impaired by aging, likely due to vascular basement membrane thickening with altered extracellular matrix components [135]. Carrying *APOE4*, which is the strongest genetic risk factor for late-onset AD [136,137], also alters basement membrane formation in *APOE4*-targeted replacement mice, which likely disturbs perivascular clearance of Aβ40 [138]. Thus, further studies should determine how basement membranes in the neurovascular unit are affected during AD and how their alteration contributes to disease pathogenesis by impacting Aβ elimination along the cerebrovasculature.

## 5. Transport of Aβ across the BBB

During Aβ drainage through the lymphatic or glymphatic pathways, a portion of Aβ could be degraded in extracellular space by diverse proteases, including neprilysin and insulin-degrading enzymes [139]. Cells at the neurovascular unit also have the ability to endocytose Aβ and clear it through lysosomal degradation [140]. Furthermore, endothelial cells likely mediate Aβ transport across the BBB by expressing several receptors and transporters, such as the low-density lipoprotein receptor-related protein 1 (LRP1), P-glycoprotein (P-gp), and the receptor for advanced glycation end products (RAGE).

Reduced LRP1 expression in brain microvessels [141] and endothelial cells [142] was observed in AD patients and amyloid mouse models. Importantly, endothelial cell-specific deletion of LRP1 has been shown to disturb Aβ clearance, resulting in the aggravated amyloid pathology in mouse models [143]. Thus, endothelial cells may possess the active transport system of Aβ across the BBB mediated by LRP1, as reported previously [141,142], although another study failed to confirm the presence of this mechanism [144]. In the endothelial cells, internalized Aβ through LRP1 on the abluminal side may either be transported into the lysosome for degradation or moved to the luminal side by transcytosis, depending on conditions [144,145,146,147,148], although further studies are required to confirm this. In addition to LRP1, P-gp is also likely involved in Aβ clearance at the BBB. P-gp is an ATP-dependent efflux transporter that is predominantly expressed in epithelial cell types, including the luminal surface of the endothelial cells in the BBB [149]. An animal study has shown that P-gp deficiency suppresses Aβ clearance and increases brain Aβ deposition [150], while P-gp expression was reduced near amyloid plaques in an amyloid mouse model [151]. In capillaries isolated from mouse brains, P-gp degradation is facilitated by the exposure with Aβ40, thereby reducing P-gp transport activity [152]. Consistent with the findings in animals, cerebrovascular expression of P-gp is inversely correlated with Aβ plaque numbers in individuals without dementia [153]. In addition, a PET study using (R)-[^11^C] verapamil has demonstrated that P-gp transporter function at the BBB is compromised in AD patients [154]. Thus, downregulations of LRP1 or P-gp in endothelial cells during AD progression are predicted to further exacerbate parenchymal Aβ accumulation by decreasing Aβ clearance from the brain. Indeed, pharmacological approaches to increase LRP1 [155] or P-gp [156] in brain capillaries likely facilitate Aβ clearance, thereby reducing brain Aβ levels in amyloid mouse models.

RAGE, an immunoglobulin superfamily member, functions as a receptor for a series of ligands including Aβ [157]. While RAGE is expressed in almost all brain cell types, including endothelial cells, vascular smooth muscle cells, microglia, astrocytes, and neurons [158], a significant increase in endothelial RAGE immunoreactivity was observed in postmortem AD brains compared to controls [159]. Interestingly, RAGE is known to mediate the entry of circulating Aβ into the brain across the BBB. Administration with an anti-RAGE antibody, soluble RAGE, or a RAGE-specific inhibitor suppressed the RAGE-mediated influx of peripheral Aβ40 and Aβ42, which ameliorated brain Aβ deposition in mouse models [160,161]. On the other hand, other members of endothelial ATP-dependent efflux transporters such as ABCG2 [162,163] and ABCG4 [163] have been shown to prevent Aβ entry from the blood flow into the brain across the BBB. Although downregulations of those efflux transporters at the BBB may accelerate parenchymal Aβ accumulation in physiological conditions, it remains unclear as to what extent this mechanism contributes to the AD pathogenesis, considering that BBB integrity may be compromised during aging and AD.

## 6. Summary and Perspective

**Figure 3:**
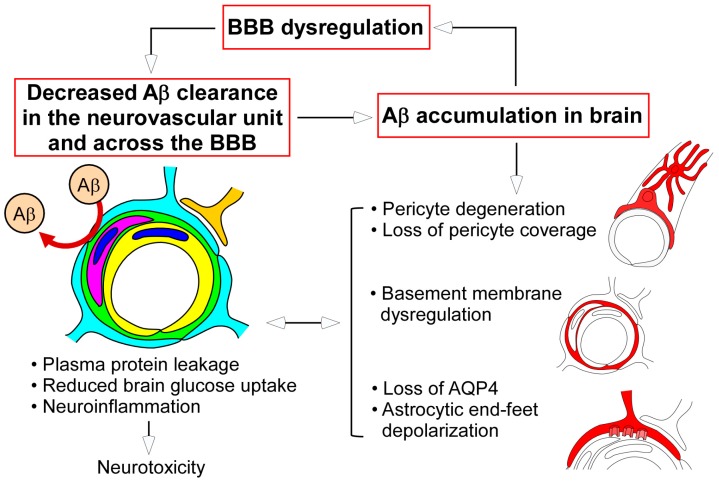
Proposed model for BBB dysfunction in AD pathogenesis. Dysregulation of the neurovascular unit (e.g., diminished endothelial transport, loss of tight junction (TJ) integrity, basement membrane disorganization, pericyte degeneration, and astrocyte depolarization) is induced during Alzheimer’s disease (AD) progression, which is particularly associated with brain Aβ accumulation. These alterations, in turn, contribute directly or indirectly to the disturbed Aβ clearance in the neurovascular unit and across the BBB, thus setting up a vicious cycle in AD pathogenesis. In parallel, plasma protein leakage, reduced brain glucose uptake, and neuroinflammation caused by BBB damage may lead to further cellular toxicity, making neurons more susceptible to AD pathologies.

It is increasingly evident that aging, cerebrovascular damage, and/or Aβ accumulation can initiate BBB dysregulation by affecting multiple components of the neurovascular unit. Disturbing BBB homeostasis not only causes neuronal damage, but also compromises Aβ clearance at the neurovascular unit, therefore likely resulting in a vicious cycle between Aβ accumulation and BBB dysfunction during AD progression (Figure 3). While BBB disruption is often detected in AD patients, it is unclear whether it is a specific feature of AD. In this regard, further efforts using larger prospective cohorts should be devoted to defining how BBB function is altered before AD onset and during disease progression, and how the alteration is correlated with AD pathologies, including senile plaque and neurofibrillary tangle formations. As discussed in the above section, one of the challenges is that the sensitivity and specificity of current techniques may not be robust enough to reliably evaluate BBB function in human cohorts. Although the further optimization of brain imaging techniques and the development of novel biomarkers to evaluate BBB function might be critical, the combination of those approaches would help to overcome this limitation. In addition, the re-evaluation of BBB pathology would be necessary by comprehensively investigating different regions of postmortem brains from cognitively healthy individuals and patients with MCI, AD, and other neurodegenerative diseases. Verifying the methodology for histological and biochemical assessments of BBB function and stratifying vascular contributions might also be required.

Given that risk factors for both AD and atherosclerotic/cardiovascular diseases significantly overlap [164,165,166], it might be important to explore vascular-mediated inflammation in AD pathogenesis. In addition to maintaining endothelial barrier formation, the BBB also serves as an interface to link the peripheral immune system to that in the CNS [167,168,169]. When cerebrovascular endothelial cells and circulating leucocytes are activated as a part of immune responses, the expression of adhesion molecules and chemoattractant productions are upregulated in those cells, thereby facilitating the invasion of circulating immune cells into brain parenchyma across the BBB [9,170]. The infiltrated immune cells likely lead to structural alterations of the BBB through the production and secretion of proinflammatory cytokines, reactive oxygen species, and active proteases. Reciprocal activation of cells at the neurovascular unit, in particular glial cells, and their production of cytotoxic mediators may also influence the BBB, further sustaining endothelial inflammation. While leukocytes such as lymphocytes, monocytes, and neutrophils likely penetrate the BBB and traffic into the brain in AD [171], neutrophil depletion has been shown to improve cognitive function and reduce AD-related pathology in amyloid model mice [172]. Thus, targeting vascular inflammation and/or leukocyte trafficking through the BBB may have therapeutic potential in AD.

In conclusion, despite accumulating evidence suggesting the link between BBB dysregulation and AD pathogenesis, how BBB alteration contributes to the overall pathogenic cascades of AD has not yet been determined. A greater understanding of how BBB dysfunction is causatively or consequently related to AD pathogenesis could allow us to develop the diagnostic and therapeutic strategies targeting BBB for this devastating disease. Further comprehensive studies that consider both the multiple functions of the BBB and the associated complexities of AD development and progression are needed.

## Abbreviations and Acronyms

5XFAD	5 familial AD mutations
ABCG2	ATP-binding cassette sub-family G member 2
ABCG4	ATP-binding cassette sub-family G member 4
AD	Alzheimer’s disease
AQP4	Aquaporin 4
Aβ	Amyloid-β
BBB	Blood-brain barrier
CA1	Cornu ammonis 1
CAA	Cerebral amyloid angiopathy
CNS	Central nervous system
CSF	Cerebrospinal fluid
CT	Computed tomography
ELISA	Enzyme-linked immunosorbent assay
GFAP	Glial fibrillary acidic protein
GLUT1	Glucose transporter 1
HPLC	High performance liquid chromatography
HUVEC	Human umbilical vein endothelial cell
ISF	Interstitial fluid
JAM	Junctional adhesion molecule
LAM	Leukocyte adhesion molecule
LRP1	Low-density lipoprotein receptor-related protein 1
MAGUK	Membrane-associated guanylate kinases
MCI	Mild cognitive impairment
MRI	Magnetic resonance imaging
mRNA	Messenger RNA
PDGFRβ	Platelet-derived growth factor receptor β
PET	Positron emission tomography
P-gp	P-glycoprotein
RAGE	Receptor for advanced glycation end products
TJ	Tight junction
VE-cadherin	Vascular endothelial-cadherin
ZO-1	Zonula occludens-1
